# Genetic Spectrum of *ABCA4*-Associated Retinal Degeneration in Poland

**DOI:** 10.3390/genes10120959

**Published:** 2019-11-21

**Authors:** Anna M. Tracewska, Beata Kocyła-Karczmarewicz, Agnieszka Rafalska, Joanna Murawska, Joanna Jakubaszko-Jablonska, Małgorzata Rydzanicz, Piotr Stawiński, Elżbieta Ciara, Muhammad Imran Khan, Arjen Henkes, Alexander Hoischen, Christian Gilissen, Maartje van de Vorst, Frans P. M. Cremers, Rafał Płoski, Krystyna H. Chrzanowska

**Affiliations:** 1 DNA Analysis Unit, ŁUKASIEWICZ Research Network–PORT Polish Center for Technology Development, 54-066 Wrocław, Poland; 2 Children’s Memorial Health Institute, 04-730 Warsaw, Poland; b.kocyla-karczmarewicz@ipczd.pl (B.K.-K.); e.ciara@ipczd.pl (E.C.);; 3 Department of Ophthalmology, Wrocław Medical University, 50-556 Wrocław, Poland; agnieszka.rafalska@student.umed.wroc.pl (A.R.); jjj@ookspektrum.nazwa.pl (J.J.-J.); 4 Department of Ophthalmology, University Clinical Centre, 80-214 Gdańsk, Poland; asiarochna@wp.pl; 5 Department of Paediatric Traumatology and Emergency Medicine, Wrocław Medical University, 50-345 Wrocław, Poland; 6 SPEKTRUM Ophthalmology Clinic, 53-334 Wrocław, Poland; 7 Department of Medical Genetics, Medical University of Warsaw, 02-106 Warsaw, Poland; malgorzata.rydzanicz@wum.edu.pl (M.R.); piotr.stawinski@wum.edu.pl (P.S.); rploski@wp.pl (R.P.); 8 Department of Human Genetics, Radboud university medical center, PO Box 9101, 6500 HB Nijmegen, The Netherland; mimranmani@gmail.com (M.I.K.); arjenhenkes@hotmail.com (A.H.); alexander.hoischen@radboudumc.nl (A.H.); maartjevandevorst@hotmail.com (M.v.d.V.); frans.cremers@radboudumc.nl (F.P.M.C.); 9 Donders Institute for Brain, Cognition and Behavior, Radboud University Medical Center, PO Box 9104, 6500 HE Nijmegen, The Netherlands; christian.gilissen@radboudumc.nl; 10 Department of Internal Medicine and Radboud Center for Infectious Diseases (RCI), Radboud University Medical Center, P.O. Box 9101, 6500 HB Nijmegen, The Netherlands

**Keywords:** *ABCA4*, Stargardt disease, cone-rod dystrophy, retinitis pigmentosa, inherited retinal disorders

## Abstract

Mutations in retina-specific ATP-binding cassette transporter 4 (*ABCA4*) are responsible for over 95% of cases of Stargardt disease (STGD), as well as a minor proportion of retinitis pigmentosa (RP) and cone-rod dystrophy cases (CRD). Since the knowledge of the genetic causes of inherited retinal diseases (IRDs) in Poland is still scarce, the purpose of this study was to identify pathogenic *ABCA4* variants in a subgroup of Polish IRD patients. We recruited 67 families with IRDs as a part of a larger study. The patients were screened with next generation sequencing using a molecular inversion probes (MIPs)-based technique targeting 108 genes involved in the pathogenesis of IRDs. All identified mutations were validated and their familial segregation was tested using Sanger sequencing. In the case of the most frequent complex allele, consisting of two variants in exon 12 and 21, familial segregation was tested using restriction fragment length polymorphism (RFLP). The most prevalent variant, a complex change c.[1622T>C;3113C>T], p.[Leu541Pro;Ala1038Val], was found in this cohort in 54% of all solved *ABCA4*-associated disorder cases, which is the highest frequency reported thus far. Additionally, we identified nine families displaying a pseudo-dominant mode of inheritance, indicating a high frequency of pathogenic variants within this population.

## 1. Introduction

Inherited retinal diseases are genetic disorders affecting the retina. A specific subgroup of these disorders is caused by mutations in a gene encoding retina-specific ATP-binding cassette transporter 4 (*ABCA4*). This enzyme displays flippase activity towards retinoid substrates, enabling influx from the intradiscal membrane or extracellular membrane in rods [1]. *ABCA4* is also expressed in the retinal pigment epithelium (RPE) [2]. Mutations disrupting this enzyme result in lipofuscin accumulation [3], which is damaging to photoreceptors and the RPE. Pathogenic alterations in the corresponding gene are responsible for various retinal dystrophies with macular involvement, mostly following an autosomal recessive inheritance pattern.

Stargardt disease 1 (STGD1, Mendelian Inheritance in Man (MIM) #248200), initially described in 1909 by a German ophthalmologist, is a macular dystrophy. In most cases, the inheritance follows an autosomal recessive pattern. In 1997, its inheritance was ascribed by Allikmets et al. to mutations in the *ABCA4* gene [4]. Although mutations in *ABCA4* cause over 95% of STGD, patients harbouring variants in this gene may also display different phenotypes: cone-rod dystrophy 3 (CRD3, MIM #604116), and retinitis pigmentosa 19 (RP19, MIM #601718) [5]. The model devised by Magueri et al. in 2000 divided *ABCA4* mutations into three categories: ‘mild’, ‘moderate’ and ‘severe’, explaining the spectrum of different phenotypes by different variant combinations [6]. There have also been reports of *ABCA4* dominant heterozygous mutations causing age-related macular degeneration (AMD, MIM #153800) [7]. However, it is hypothesised now that this entity may indeed be very late-onset Stargardt disease and that very mild and common hypomorphic alleles acting in trans contribute to the pathogenicity [8]. Up until recently, hypomorphic variants, such as p.(Asn1868Ile), were significantly underestimated in respect of the pathogenicity of *ABCA4*-associated disorders [8,9]. Additionally, deep-intronic changes were discovered earlier to have an impact on the disease when acting in trans with pathogenic alterations [10,11,12]. Over a thousand pathogenic variants have been described so far [13]. Causality and penetrance of some are still the subject of a debate [14,15].

Complex alleles in *ABCA4* have been known since the discovery of its connection with retinal diseases. Variants c.[1622T>C;3113C>T], p.[Leu541Pro;Ala1038Val] found in *cis* were previously described to be present in 33% of Polish patients. Population frequency was estimated to be 0.42% [16]. However, the broader context of *ABCA4* mutations in Poland, especially in the case of retinitis pigmentosa patients, has never been tested. Herewith, we performed molecular inversion probes (MIPs) analysis using panel of 108 genes on over two hundred inherited retinal disease (IRD) families to uncover the genetic population characteristics of IRD in Poland.

## 2. Materials and Methods

This study was approved by the local Bioethics Committee. We enrolled over two hundred families in a larger study designed to uncover the genetic causes of hereditary retinal degeneration in Poland. Informed consent adhering to the tenets of the Declaration of Helsinki was received from all participating affected individuals and unaffected family members. In addition, a Data Processing Agreement with a clause according to the General Data Protection Regulation EU Act was obtained.

A detailed medical history was obtained, and a full ophthalmologic examination was performed for all study participants. Clinical evaluation, when feasible, included best-corrected Snellen visual acuity (BCVA), colour vision, visual field test, dilated ophthalmoscopy, digital fundus photography, autofluorescence imaging, spectral-domain optical coherence tomography (SD-OCT), fluorescein angiography and electrophysiological assessment.

Genomic DNA was isolated from peripheral blood from patients and their relatives by automated method on a MagNA Pure 24 System, (Roche, Basel, Switzerland) or QIASymphony DSP DNA Mini/Midi kit on a QIASymphony robot or via manual QIAAmp Blood DNA kit (Qiagen, Hilden, Germany). DNA concentration was assessed with a Quant-iT™ dsDNA Assay Kit, broad range, using a NanoDrop 3300 spectrofluorometer (Thermofisher Scientific, Waltham, MA, USA). Subsequently, samples were screened using a molecular inversion probes (MIPs) technique targeting 108 genes involved in the pathogenesis of inherited retinal disorders (IRDs), as described elsewhere [17]. Briefly, over 6000 MIPs covering the regions of interest were designed using an in-house software (Department of Human Genetics, Radboud University Nijmegen Medical Center) and manually verified. The probes were phosphorylated and pooled together. Careful rebalancing experiments were performed to ensure uniform coverage. We prepared the libraries using Ampligase DNA ligase (Thermofisher Scientific, Waltham, MA, USA) and HemoKlenTaq polymerase (New England Biolabs, Ipswich, MA, USA) for gap filling. Polymerase chain reaction (PCR) was performed with high fidelity iProof Master Mix (Biorad, Hercules, CA, USA). We tested the hybridisation efficiency using real-time PCR to empirically determine the optimal number of amplification cycles in each separate pool. Subsequently, PCR products were verified on a 2% agarose gel, pooled and purified using Agencourt AMPure magnetic beads (Beckman-Coulter, Brea, CA, USA). The purity and size of the product was checked on a Fragment Analyzer with High Sensitivity NGS (next-generation sequencing) Fragment Analysis Kit (DNF-474; Agilent Technologies, Santa Clara, CA, USA). Libraries were quantified using a NanoDrop 3300 spectrofluorometer with the Quant-iT™ dsDNA Assay Kit, high sensitivity (Thermofisher Scientific, Waltham, MA, USA). The pools were paired-end sequenced (2× 100 bp) with a Rapid Run Mode on a HiSeq 1500 (Illumina, San Diego, CA, USA) according to the manufacturer’s protocol. For each run, custom sequencing primers were added at a final concentration of 0.5 µM. Raw sequence readouts were analyzed with bcl2fastq software (Illumina) to generate fastq format reads. After the quality control step (including adapter trimming and low quality reads removal), reads were aligned to the hg38 reference genome with Burrows-Wheeler Aligner (BWA; [18]), and processed further by Picard [19] and Genome Analysis Toolkit [20].

We performed secondary analysis of fastq files with the SeqNext module of SeqPilot software. The frequency filter was initially set to minor allele frequency (MAF) <0.5% in public databases, following manual identification of causative variants appearing with higher frequency in global populations. All identified alterations were validated using Sanger sequencing. We recruited family members to perform segregation analysis. Genetic material was self-collected with buccal swabs by family members and DNA was isolated with the QIASymphony Investigator Kit (Qiagen, Hilden, Germany). We have confirmed familial segregation using Sanger sequencing in all amplicons except for the most common variants inherited together as a complex allele: c.1622T > C, p.(Leu541Pro) and c.3113C > T, p.(Ala1038Val), where we used restriction enzymes *Tsp*RI and *Bse*YI, respectively (New England Biolabs, Ipswich, MA, USA). We subjected novel splice variants to scrutiny using four *in silico* prediction programs which are incorporated into AlaMut Visual Splicing Effects module SpliceSiteFinder-like (SSFL), MaxEntScan (MES), NNSPLICE, GeneSplicer (Biosoftware, 2014; Interactive Biosoftware, Rouen, France).

## 3. Results

### 3.1. Clinical Testing Results

Out of 67 individuals analysed in this study, 50 probands were diagnosed with STGD, 12 were suffering from RP, three were suffering from CD/CRD and two probands had an unsure diagnosis. Age of onset presented a wide spectrum from 4 to 44 years old (median = 9), and age at diagnosis was 7–62 years old (median = 11) (Table 1). At the time of recruitment, patients ranged in age from 8 to 63 years old (median = 31). There was no difference in median age of onset between RP and STGD1 patients.

The most common clinical presentation of STGD1 patients was RPE atrophy and pigment clumping in the macula. Characteristic fundus flecks were noted in 28% of patients. The dark choroid sign on fluorescein angiography was present in 46% of cases. Fundus autofluorescence showed central areas of hyperautofluorescence in all tested subjects. Multifocal electroretinography (mfERG) proved to be the most useful electrophysiological test with reduced response from the central 10 degrees confirming the diagnosis. mfERG pattern varied greatly in STGD1 subjects, ranging from a normal response to rod and/or cone abnormalities, which correlated poorly with clinical presentation. The best corrected distance visual acuity (BCVA) was 0.1 in the majority of patients and they mostly retained reading ability. All 12 of the patients diagnosed with RP experienced visual deterioration and nyctalopia before the age of 10. They mostly presented with the classic triad of arteriolar attenuation, optic nerve pallor and bone spicules in the peripheral retina. The disease led to severe visual impairment and legal blindness in the majority of cases—BCVA was limited to light perception in one third of cases and hand movement in another one third of cases. Ten out of 12 patients lost reading ability.

**Figure 1:**
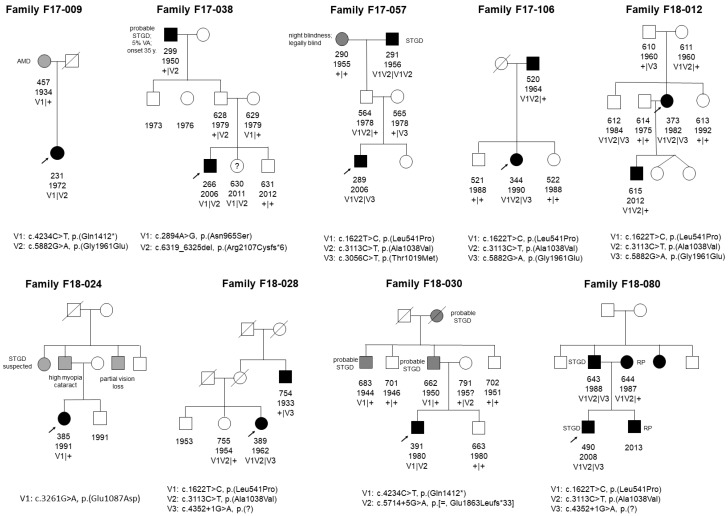
Pedigrees of nine families with pseudo-dominant inheritance pattern. Various phenotypes are present within families. Only variants detected in probands were sequenced in family members. Patient 385 remains unsolved. AMD = age-related macular dystrophy; STGD = Stargardt disease; RP = retinitis pigmentosa; VA = visual acuity; V = variant.

Additionally, we detected nine families displaying a pseudo-dominant mode of inheritance in this group. Their corresponding pedigrees with family members available for co-segregation are depicted in Figure 1. Segregation analysis was not possible in three cases, since we lost contact with the families.

### 3.2. Genetic Testing Results

**Figure 2:**
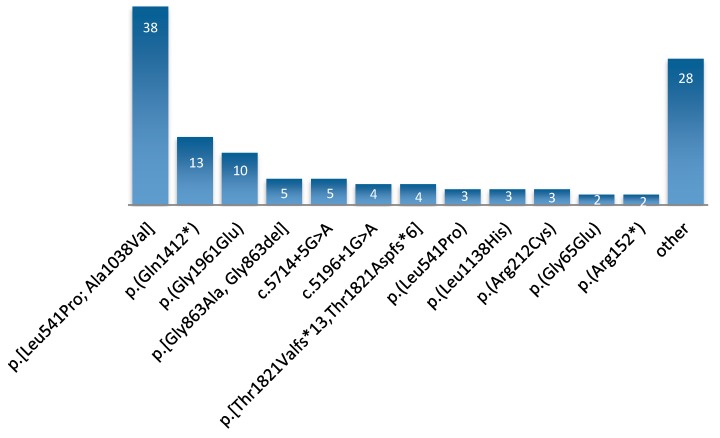
Number of alleles for the most common variants. The most prevalent, complex allele was found on 38 chromosomes. Its component, c.1622T > C, p.(Leu541Pro), was identified independently on three additional alleles. Unique causative alterations present on one chromosome in a single patient are combined within “other”.

After secondary in silico analysis, we found *ABCA4* variants in 63 probands. Four additional STGD patients received no genetic diagnosis. Two of them underwent whole exome sequencing (WES) with no results. The complex allele c.[1622T>C;3113C>T], p.[Leu541Pro;Ala1038Val] was present in 32 individuals suffering from *ABCA4*-associated disorders (six of them homozygous), which makes it the most prevalent allele in the Polish population (50.7% of all cases in subgroup with *ABCA4* variants; 38/123 pathogenic alleles; MAF = 0.31) (Figure 2, Table 1, Table S2). Additionally, we found a second variant from the complex allele independently in three cases. All cases taken together represented the whole spectrum of *ABCA4*-related diagnoses. All identified mutations are shown in Table S2.

Three mutations found in this cohort were thus far unreported. We identified c.5899-3T>G, p.?; c.66G>A, p.[=,?] and a copy number variant (CNV) encompassing exons 18–50, whose breakpoint could not be determined due to ending downstream of the *ABCA4* gene (c.(2653+1_2654-1)_(*1_?)del, p.(Gly885Valfs*71); [21]). All detected mutations are shown in Table 1 and Table S1. The corresponding MAFs and classifications are shown in Table S2.

## 4. Discussion

We have hereby proven our hypothesis that the spectrum of *ABCA4* alterations in Poland differs from Western populations. Three novel changes were identified—one was a CNV and two represented splice site variants (the latter two lately published in the Human Genome Mutation Database). Interestingly, one of them was a silent type, affecting the last nucleotide in exon 1 (p.Lys22=). All splice prediction algorithms showed almost completely abolished donor splice sites (SSFL: −100%, MES: −79%, NNSPLICE: −100%, GeneSplicer: −100%). In case of c.5899-3T > G the acceptor splice site estimates were also severely diminished in all except one predictor (SSFL: −6%, MES: −100%, NNSPLICE: −100%, GeneSplicer: −100%). Both patients had a ‘severe’ variant on the other allele inherited from their fathers (p.(Arg152*) and p.[Leu541Pro;Ala1038Val]). Individual 424, suffering from RP, has inherited c.5899-3T > G from the mother and 325, who has STGD1, received the (p.Lys22=) allele. Therefore, according to previous classifications, we may assume that the first one represents a null mutation and the latter results in a moderate effect. However, midigene splice site assays, such as the ones performed by Sangermano et al. [22], are necessary to determine their actual effects.

CNVs in the *ABCA4* gene are not common events [11,23,24]. Patient 302, who harboured a large deletion on one allele, had classic Stargardt disease with juvenile age of onset (nine years old). The presence of a CNV was strongly suggested by the fact that a single heterozygous mutation, c.2588G > C, p.[Gly863Ala, Gly863del], was accompanied by the seemingly homozygous c.5603A > T, p.(Asn1868Ile) and upon segregation, the father appeared to harbour both changes heterozygously, and the mother had none. This suggested a local loss of heterozygosity. A CNV analysis using the SeqNext CNV module revealed a deletion of exons 18–50, which was subsequently confirmed in a smMIPs(single-molecule molecular inversion probes)-based whole *ABCA4* gene sequencing study [21]). Most probably the mother is a carrier of this variant. Unfortunately, since the deletion stretches further downstream of the *ABCA4* gene, its breakpoints could not be determined and thus segregation testing of this variant was not performed.

Only eight patients in our group were homozygous: six for the aforementioned complex allele, one for p.(Gln1412*)—the second most common allele in this cohort, present on 13 chromosomes—and one for c.5196 + 1G > A. This is in line with the general outbred profile of the Polish population. Notably, individual 333 carrying c.5196 + 1G > A on both alleles was born of a consanguineous union of second degree cousins, which is a practice uncommon within this society. Additionally, one of patient’s 289 affected grandparents was homozygous for the p.[Leu541Pro;Ala1038Val] complex variant (consanguinity not known). In family F18-002, where two pairs of siblings intermarried, second degree cousins displayed the same combination of p.(Gln1412*) and p.(Leu541Pro) variants, which resulted in the RP phenotype.

RP patients almost invariably displayed only alleles deemed “severe”, whereas the STGD1 phenotype could be ascribed to a broader spectrum of alterations, suggesting genetic modifiers. The only RP patients who were not solved with severe mutations (419 and 420) harboured a mild change and variants of unknown significance (VUS). Individual 419 had p.(Gly1961Glu) and p.(Glu471Lys) VUS, which segregated within the family. The p.(Glu471Lys) variant has been described in the literature before, both as a VUS and a likely pathogenic variant. Initially, Allikmets et al. reported it as an AMD-associated variant [7], which may indicate that it exerts a very mild effect on protein activity. Lately, it was reported in a phenotype called bull’s-eye maculopathy [25]; however, in both cases, no deep intronic variants or CNV screening was performed, indicating that it may be a ‘passenger’ mutation linked with an unknown pathogenic variant. It is a matter of debate whether patient 419 could be considered as solved, despite having two segregating *ABCA4* variants and no other variant within the 108 genes studied. Further studies involving broader analysis including deep intronic variants in *cis*, possibly to identify a third, severe mutation, are required. Similarly, individual 420 had only single-allelic VUS, p.(Pro196Leu), which is most likely not a causative mutation. In this RP patient, the genetic cause most likely lies within another gene. Four additional STGD patients had no candidate *ABCA4* variants. Two of them underwent WES, but the results were negative. These individuals may carry deep intronic variants or CNVs in *ABCA4*, which should be elucidated in the next step.

Classical Stargardt disease was also displayed by patient 385, who was a carrier of the heterozygous c.3261G > A, p.(Glu1087Asp) variant. Patient 418 was another single allele carrier who first noticed disease symptoms at the age of 27. A heterozygous VUS, c.5887C > T p.(Arg1963Cys), was identified with no counterpart on the other chromosome. Despite her rather advanced age (63), this individual showed mild symptoms and still retained some vision in her left eye. Both of these patients have not yet been tested for deep intronic variants.

We revealed phenotype discordance in four families. Discordant siblings, carrying the same set of mutations but showing no phenotype, were invariably male. In one case, an eight-year-old individual with c.[1622T>C;3113C>T], p.[Leu541Pro;Ala1038Val] and c.2588G > C, p.[Gly863Ala, Gly863del] may not have yet developed the symptoms (his sister started noticing visual problems at the age of nine). Patient 235 had two brothers with the same pathogenic variants: c.[1622T>C;3113C>T], p.[Leu541Pro; p.Ala1038Val] and c.5714+5G > A, p.[=, Glu1863Leufs *33]. Her age of onset was seven, and the affected younger brother was 13 years old. The other brother was already 26 years old and displayed no phenotype. The same situation was seen in the pseudo-dominant family of patient 373; however, here the difference between the ages of onset was smaller. The proband, carrying c.[1622T>C;3113C>T], p.[Leu541Pro;Ala1038Val] and c.5882G > A, p.(Gly1961Glu) mutations first had symptoms at 28. Her brother was still asymptomatic at 35. The last family harboured the c.4234C > T, p.(Gln1412*) c.1654G > A, p.(Val552Ile) combination. In this case, no intronic variants or CNVs were found in a parallel study [21]). Age of onset of the proband was 22, whereas his brother showed no symptoms at 34. These inconsistencies may indicate the presence of a genetic modifier within these families. Further studies are required to uncover possible variants influencing the penetrance and expressivity of the disease.

It is striking how extremely common the p.[Leu541Pro;Ala1038Val] allele is in the populations of Mid-Eastern Europe. It was first found in 1997 by Allikmets et al. upon the identification of *ABCA4* variants as a cause of Stargardt disease. Subsequently, this combination was detected in many other families. The first suggestion of a German origin founder effect of the p.[Leu541Pro;Ala1038Val] allele was made by Rivera in 2000 [26], when it was found among STGD1 and AMD patients with a frequency of 12.7%. However, more recent publications indicate that the high frequency in Germany was rather due to migrations from Eastern Europe, where this allele is more common. The Hungarian population was found to display a frequency of 28% among STGD patients [27]. Finally, Scieżyńska et al. tested 93 unrelated Polish STGD and CRD patients and detected this allele in 33 individuals [16]. The population frequency was estimated as 0.42%. In the current in-house database, which now contains data from 5007 samples, this allele appears 33 times (0.63%), and p.(Ala1038Val) alone appears twice. This means that crossing-over does indeed occur between these two linked loci, albeit it is not a frequent event.

In a recent publication, the three-dimensional (3D) structure of ABCA4 was devised and structural in silico tools were used to link the functional effects of mutations to the related phenotypes [28]. The pathogenic effect of p.(Leu541Pro), residing in extracellular domain 1, was reported to result from misfolding. Garces et al. described this variant earlier as having a negative impact on the ATP-binding capacity and abolishing of retinal-stimulated ATP hydrolysis [29]. The second variant of the complex allele, p.(Ala1038Val), located in nucleotide binding domain 1, seemed to be a milder alteration. Nevertheless, it still contributed to the severe outcome [13,29,30].

Out of the 67 patients in this group, nine came from families showing a clear pseudo-dominant pattern, either with full or incomplete penetrance (Figure 1). Pseudo-dominance in this gene has been reported many times [6,31,32]. This plays a crucial role, especially in cases where clinical phenotype cannot be easily determined. For instance, cone-rod dystrophy is a disease that can be inherited in both autosomal recessive and dominant mode. Wrong conclusions about risks for family members can be drawn if misinterpreted in the absence of genetic diagnosis.

Four patients with STGD could not be genetically solved. Despite performing additional WES in two cases, no causative mutations could be found in these individuals. However, neither MIP analysis nor WES is able to reliably detect large deletions, duplications, inversions or other changes of this kind. Although rare, CNVs and large rearrangements in *ABCA4* could be responsible for the lack of genetic diagnosis.

The frequency of pathogenic *ABCA4* variants in the Polish population is relatively high (Table S2). According to Magueri’s hypothesis, compound heterozygous combinations of a mild and a severe variant would cause STGD1, moderate and severe variants would cause CRD, and severe variants on both chromosomes would result in RP [6]. Lately, a third category of very mild variants has been discovered. With the recent emergence of proof of pathogenicity in hypomorphic and deep intronic variants, a doctor has to be very careful in establishing the risks for future offspring and grandchildren of the affected individuals. For example, children of a patient with RP, due to the presence of two severe alleles in the parent, would have a different lifetime risk of developing retinal degeneration than in case of ‘regular’ recessive Mendelian disorders. A 100% chance of inheriting a severe allele from an affected parent by such a child would meet a possibility of getting one of the very mild common alleles from the unaffected parent. Combined together, these variants may appear in the population with a frequency even higher than 10%. Even though there is some non-penetrance, this risk would still be higher than in other rare diseases. This has a great impact on genetic counselling. Therefore, clinical geneticists should be thoroughly informed before giving reproductive advice to the patients and their family members.

Taken together, our results show that 39 individuals (58% of our cohort) could be genetically solved with just 11 of the most frequent variants depicted in Figure 2. Since next-generation sequencing is still not popular in Poland due to its costs, it may be an indication to screen for these mutations in the first instance. Especially with emerging new therapy prospects, patients have the right to a proper genetic diagnosis.

## Figures and Tables

**Table 1:** Genetic and demographic data of patients suffering from retina-specific ATP-binding cassette transporter 4 (*ABCA4*)-related disorders. CD = cone dystrophy; CRD = cone-rod dystrophy; RP = retinitis pigmentosa; STGD1 = Stargardt’s dsease type 1

Sample ID	Family ID	Year of birth	Sex	Diagno-sis	Age of onset	Age at dia-gnosis	Age at exami-nation	Allele 1		Allele 2	
								Nucleotide level	Protein level	Nucleotide level	Protein level
225	F17-003	1983	F	STGD1	7.0	7.0	34	c.[1622T>C;3113C>T]	p.[Leu541Pro;Ala1038Val]	c.634C>T	p.(Arg212Cys)
229	F17-007	1988	F	STGD1	7.0	9.0	29	c.4537dup	p.(Gln1513Profs*42)	c.5461-10T>C	p.[Thr1821Valfs*13,Thr1821Aspfs*6]
230	F17-008	1954	M	STGD1	44.0	62.0	63	c.[1622T>C;3113C>T]	p.[Leu541Pro;Ala1038Val]	c.5603A>T	p.(Asn1868Ile)
231	F17-009	1972	F	STGD1	14.0	16.0	45	c.4234C>T	p.(Gln1412*)	c.5882G>A	p.(Gly1961Glu)
235	F17-010	1989	F	STGD1	7.0	11.0	28	c.[1622T>C;3113C>T]	p.[Leu541Pro;Ala1038Val]	c.5714+5G>A	p.[=,Glu1863Leufs*33]
239	F17-014	1984	M	STGD1	30.0	31.0	33	c.194G>A	p.(Gly65Glu)	c.2588G>C	p.[Gly863Ala,Gly863del]
240	F17-015	1980	M	STGD1	8.0	10.0	37	c.[1622T>C;3113C>T]	p.[Leu541Pro;Ala1038Val]	c.[1622T>C;3113C>T]	p.[Leu541Pro;Ala1038Val]
247	F17-022	1990	F	STGD1	13.0	23.0	27	c.194G>A	p.(Gly65Glu)	c.5882G>A	p.(Gly1961Glu)
252	F17-025	2009	M	STGD1	7.0	8.0	8	c.[1622T>C;3113C>T]	p.[Leu541Pro;Ala1038Val]	c.1622T>C	p.(Leu541Pro)
253	F17-026	2006	M	STGD1	8.5	9.0	11	c.[1622T>C;3113C>T]	p.[Leu541Pro;Ala1038Val]	c.2041C>T	p.(Arg681*)
255	F17-028	2001	F	STGD1	7.0	7.5	16	c.5684_5685delTG	p.(Leu1895Argfs*16)	c.5882G>A	p.(Gly1961Glu)
264	F17-036	1999	M	STGD1	14.0	16.0	18	c.[1622T>C;3113C>T]	p.[Leu541Pro;Ala1038Val]	c.4462T>C	p.(Cys1488Arg)
266	F17-038	2006	M	STGD1	9.0	9.5	11	c.2894A>G	p.(Asn965Ser)	c.6319_6325del	p.(Arg2107Cysfs*6)
277	F17-045	2004	M	STGD1	11.0	12.0	13	c.1211C>A	p.(Ser404*)	c.5882G>A	p.(Gly1961Glu)
284	F17-052	2001	M	STGD1	10.0	16.0	16	c.454C>T	p.(Arg152*)	c.2588G>C	p.[Gly863Ala,Gly863del]
286	F17-054	1981	F	RP	8.0	11.0	36	c.2626C>T	p.(Gln876*)	c.5196+1G>A	p.(?)
289	F17-057	2006	M	STGD1	8.0	9.0	11	c.[1622T>C;3113C>T]	p.[Leu541Pro;Ala1038Val]	c.3056C>T	p.(Thr1019Met)
298	F17-062	2005	M	STGD1	8.0	9.0	12	c.710T>C	p.(Leu237Pro)	c.4234C>T	p.(Gln1412*)
302	F17-065	2004	F	STGD1	9.0	11.0	13	c.[2588G>C;5603A>T]	p.[Gly863Ala,Gly863del;Asn1868Ile]	c.(2653+1_2654-1)_(*1_?)del	p.(Gly885Valfs*71)
305	F17-068	2005	M	STGD1	11.0	11.0	12	c.[1622T>C;3113C>T]	p.[Leu541Pro;Ala1038Val]	c.4139C>T	p.(Pro1380Leu)
309	F17-072	1987	F	STGD1	8.0	8.5	30	c.[1622T>C;3113C>T]	p.[Leu541Pro;Ala1038Val]	c.4234C>T	p.(Gln1412*)
314	F17-077	2003	F	STGD1	12.0	14.0	14	c.61C>T	p.(Gln21*)	c.3413T>A	p.(Leu1138His)
316	F17-079	2007	F	CD	8.0	9.5	10	c.[1622T>C;3113C>T]	p.[Leu541Pro;Ala1038Val]	c.[1622T>C;3113C>T]	p.[Leu541Pro;Ala1038Val]
318	F17-081	2009	F	STGD1	7.5	8.0	8	c.3364G>T	p.(Glu1122*)	c.4234C>T	p.(Gln1412*)
321	F17-083	2004	M	STGD1	11.5	12.0	13	c.4234C>T	p.(Gln1412*)	c.5882G>A	p.(Gly1961Glu)
325	F17-086	2004	F	STGD1	11.0	12.0	13	c.454C>T	p.(Arg152*)	c.66G>A	p.[=,?]
327	F17-088	2008	F	STGD1	7.5	8.0	9	c.[1622T>C;3113C>T]	p.[Leu541Pro;Ala1038Val]	c.4234C>T	p.(Gln1412*)
333	F17-095	1994	M	RP	10.0	11.0	23	c.5196+1G>A	p.(?)	c.5196+1G>A	p.(?)
338	F17-100	1976	F	STGD1	10.0	12	41	c.[1622T>C;3113C>T]	p.[Leu541Pro;Ala1038Val]	c.[1622T>C;3113C>T]	p.[Leu541Pro;Ala1038Val]
344	F17-106	1990	F	STGD1	7.0	19.0	27	c.[1622T>C;3113C>T]	p.[Leu541Pro;Ala1038Val]	c.5882G>A	p.(Gly1961Glu)
345	F17-107	1989	F	STGD1	18.0	20.0	28	c.[1622T>C;3113C>T]	p.[Leu541Pro;Ala1038Val]	c.5882G>A	p.(Gly1961Glu)
347	F17-109	1990	F	STGD1	16.0	20.0	27	c.[1622T>C;3113C>T]	p.[Leu541Pro;Ala1038Val]	c.5714+5G>A	p.[=,Glu1863Leufs*33]
348	F17-110	1975	F	STGD1	19.0	20.0	42	c.4234C>T	p.(Gln1412*)	c.2588G>C	p.[Gly863Ala,Gly863del]
349	F17-111	1992	M	STGD1	22.0	24.0	25	c.4234C>T	p.(Gln1412*)	c.1654G>A	p.(Val552Ile)
351	F17-113	1994	M	STGD1	9.0	9.0	23	c.2588G>C	p.(Gly863Ala)	c.5461-10T>C	p.[Thr1821Valfs*13,Thr1821Aspfs*6]
356	F17-118	1990	F	STGD1	21.0	27.0	27	c.[1622T>C;3113C>T]	p.[Leu541Pro;Ala1038Val]	c.5882G>A	p.(Gly1961Glu)
363	F18-002	1968	F	RP	7.0	11.0	50	c.4234C>T	p.(Gln1412*)	c.1622T>C	p.(Leu541Pro)
364	F18-003	1987	F	STGD1	4.0	15.0	31	c.3413T>A	p.(Leu1138His)	c.4919G>A	p.(Arg1640Gln)
370	F18-009	1975	M	RP	7.0	9.0	43	c.[1622T>C;3113C>T]	p.[Leu541Pro;Ala1038Val]	c.1622T>C	p.(Leu541Pro)
373	F18-012	1982	F	STGD1	28.0	33.0	36	c.[1622T>C;3113C>T]	p.[Leu541Pro;Ala1038Val]	c.5882G>A	p.(Gly1961Glu)
377	F18-016	1991	M	STGD1	8.0	21.0	27	c.3259G>A	p.(Glu1087Lys)	c.5714+5G>A	p.[=,Glu1863Leufs*33]
379	F18-018	1996	F	STGD1	?	21	22	c.[1622T>C;3113C>T]	p.[Leu541Pro;Ala1038Val]	c.[1622T>C;3113C>T]	p.[Leu541Pro;Ala1038Val]
385	F18-024	1995	F	CRD	12.0	19.0	23	c.3261G>A	p.(Glu1087Asp)		
389	F18-028	1962	F	RP	7.0	7	56	c.[1622T>C;3113C>T]	p.[Leu541Pro;Ala1038Val]	c.4234C>T	p.(Gln1412*)
391	F18-030	1979	M	STGD1	24.0	26.0	39	c.4234C>T	p.(Gln1412*)	c.5714+5G>A	p.[=,Glu1863Leufs*33]
397	F18-036	1963	F	RP	8.5	9.5	55	c.4793C>A	p.(Ala1598Asp)	c.5196+1G>A	p.(?)
403	F18-040	2000	F	STGD1	10.0	13.0	18	c.3413T>A	p.(Leu1138His)	c.4070C>T	p.(Ala1357Val)
407	F18-044	1957	F	RP	4.0	14	61	c.[1622T>C;3113C>T]	p.[Leu541Pro;Ala1038Val]	c.[1622T>C;3113C>T]	p.[Leu541Pro;Ala1038Val]
410	F18-047	1972	F	RP	7.0	8.0	46	c.1937+1G>A	p.(?)	c.4918C>T	p.(Arg1640Trp)
413	F18-050	1977	M	STGD1	8.0	8.5	41	c.4234C>T	p.(Gln1412*)	c.4234C>T	p.(Gln1412*)
417	F18-054	1983	M	STGD1/CRD	8.0	8.5	35	c.[1622T>C;3113C>T]	p.[Leu541Pro;Ala1038Val]	c.5461-10T>C	p.[Thr1821Valfs*13,Thr1821Aspfs*6]
418	F18-055	1955	F	STGD1/CRD	27.0	60.0	63	c.5887C>T	p.(Arg1963Cys)		
419	F18-056	1981	F	RP	5.0	18.0	37	c.5882G>A	p.(Gly1961Glu)	c.1411G>A	p.(Glu471Lys)
420	F18-057	1959	M	RP	26.0	26	59	c.587C>T	p.(Pro196Leu)		
424	F18-061	1980	M	RP	10.0	23.0	38	c.[1622T>C;3113C>T]	p.[Leu541Pro;Ala1038Val]	c.5899-3T>G	p.(?)
427	F18-064	2000	F	STGD1	5.0	10.0	18	c.[1622T>C;3113C>T]	p.[Leu541Pro;Ala1038Val]	c.1022A>G	p.(Glu341Gly)
428	F18-065	1972	F	RP	8.0	10.0	46	c.[1622T>C;3113C>T]	p.[Leu541Pro;Ala1038Val]	c.5461-10T>C	p.[Thr1821Valfs*13,Thr1821Aspfs*6]
478	F18-069	2005	F	STGD1	9.0	12.0	13	c.[1622T>C;3113C>T]	p.[Leu541Pro;Ala1038Val]	c.2588G>C	p.[Gly863Ala,Gly863del]
479	F18-070	2000	F	STGD1	7.5	8.0	18	c.[1622T>C;3113C>T]	p.[Leu541Pro;Ala1038Val]	c.634C>T	p.(Arg212Cys)
480	F18-071	2009	F	CRD	7.0	8.0	9	c.[1622T>C;3113C>T]	p.[Leu541Pro;Ala1038Val]	c.[1622T>C;3113C>T]	p.[Leu541Pro;Ala1038Val]
485	F18-075	2007	M	STGD1	9.0	9.5	11	c.[1622T>C;3113C>T]	p.[Leu541Pro;Ala1038Val]	c.634C>T	p.(Arg212Cys)
486	F18-076	2007	M	STGD1	9.0	10.0	11	c.[1622T>C;3113C>T]	p.[Leu541Pro;Ala1038Val]	c.5714+5G>A	p.[=,Glu1863Leufs*33]
490	F18-080	2008	M	STGD1	9.0	9.0	10	c.[1622T>C;3113C>T]	p.[Leu541Pro;Ala1038Val]	c.4352+1G>A	p.(?)

## Supplementary Materials

The following are available online at https://www.mdpi.com/2073-4425/10/12/959/s1, Figure S1: Family pedigrees, Table S2: Clinical and genetic characteristics of all probands.
